# Complete mitochondrial genome of *Penaeus acehensis* (Crustacea, Decapoda, Penaeidae) from Aceh province, Indonesia

**DOI:** 10.1080/23802359.2018.1495124

**Published:** 2018-08-13

**Authors:** Sinar Pagi Sektiana, M. Tahang, Sapto Andriyono, Jobaidul Alam, Hyun-Woo Kim

**Affiliations:** a Interdisciplinary Program of Biomedical, Mechanical, and Electrical Engineering, Pukyong National University, Busan, Republic of Korea;; b Aquaculture Technology Study Program, Sekolah Tinggi Perikanan, Jakarta, Indonesia;; c Brackishwater Aquaculture Development Center, Aceh, Indonesia;; d Fisheries and Marine Faculty, Universitas Airlangga, Surabaya, Indonesia;; e Department of Marine Biology, Pukyong National University, Busan, Republic of Korea

**Keywords:** Mitogenome, Dendrobranchiata, next-generation sequencing, shrimp, Indonesia

## Abstract

Penaeid shrimps are widely distributed from Indian to western Pacific Oceans and some which are economically important. In this study, we reported full mitochondrial genome of an endemic shrimp species, *Penaeus acehensis*, which inhabits exclusively in the coastal water of Aceh, Indonesia. Full length of circular mitogenome of *P. acehensis* was 15,991 bp in length, which contained 13 protein**-**coding genes, 2 rRNA genes, 22 tRNA genes, and a control region. Start codons of all protein-coding genes were ATN except for COX1 in which ACG was used. Incomplete stop codon (T- -) was found in five genes including COX2, COX3, NAD5, NAD4, and NAD4L. Among its relatives, *P. acehensis* was most closely related to *Penaeus monodon* showing 89% sequence identity in its mitogenome, which was corresponding to morphological analysis. Phylogenetic tree result showed that *P. acehensis* was clustered together with those were distributed in Indo-West Pacific region (clade II), which is distinct from Eastern Pacific region (clade I).

Although penaeid shrimps are the economically important species in Southeast Asian countries, their native populations are being seriously threatened by the careless development in the coastal area (Páez-Osuna 2001). *Penaeus acehensis* was recently identified penaeid shrimp, which is exclusively distributed in Aceh province, Indonesia (Wedjatmiko 2009). Reddish body color without transverse band and unique numbers of rostral teeth (7–8) and ventral teeth (0–5) of *P. acehensis* are the morphological characteristics distinguished from its relatives including banana shrimp (*Penaeus merguensis*) or a black tiger shrimp (*Penaeus monodon*) (Alafanta 2014, Idami 2016, Wedjatmiko 2017).

In this research, the complete mitochondrial genome sequence of *P. acehensis* was determined using the combination of NGS and conventional PCR-based cloning methods. Voucher *P. acehensis* specimens were obtained from Brackishwater Aquaculture Development Center (BADC) in Ujung Batee, Aceh province, Indonesia. Genomic DNA was extracted from the muscle using an Accuprep Genomic DNA Extraction Kit (Bioneer) according to the manufacturer’s instruction. Full mitochondrial genome sequence of *P. acehensis* was obtained by assembling five fragmental PCR products generated by degenerated primer sets designed by the multiple alignments of mitogenome sequences from its relatives. For the sequencing, all PCR products were pooled together in equal concentration and fragmented into 350 bp in length by covaris M220 (Covaris Inc.). Thruseq^®^ sample preparation kit version 2 (Illumina, USA) was used for the construction of a library and sequencing was performed using Illumina Miseq (Illumina, USA). Mothür software version 135.0 (Schloss et al. 2009) was used for pairing sequences and Geneious^®^ 11.0.2 (Kearse et al. 2012) was used for mitogenome assembly.

The total mitochondrial genome of *P. acehensis* is 15,991 bp in length (GenBank accession no. MG650292), which comprised 13 protein-coding genes, 22 transfer RNAs (RNAs), 2 ribosomal RNAs (rRNAs), and a putative control region. A + T content (71%) was higher than G + C content (29%) and the highest A + T content was observed in the putative control region (83%). Total 14 genes were located at L strand whereas the other remaining 23 genes were at H strands. Overlapping protein-coding genes were detected between ATP8 and ATP6 (7 bp), between ND4 and ND4L (19 bp). Each tRNA genes are predicted to be folded into a typical clover-leaf secondary structure except for tRNA^ser(GCT)^, which was predicted lacked D-arm. The phylogenetic trees were constructed based on minimum evolution algorithm, and *Acetes chinensis* was employed as an outgroup. Nucleotide sequence identity of *P. acehensis* ranged between 81% and 89%, and *P. monodon* was most closely related among the compared penaeid shrimps (Figure 1). Phylogenetic analysis of penaeid shrimps showed two distinct clades (Clade I and II), which implicate a geographical distribution. Shrimps inhabit in Eastern Pacific waters including *P. acehensis*, *P. monodon*, and *P. indicus* were clustered into Clade I, whereas those inhabit in Indo west Pacific waters were clustered into Clade II.

**Figure 1.**
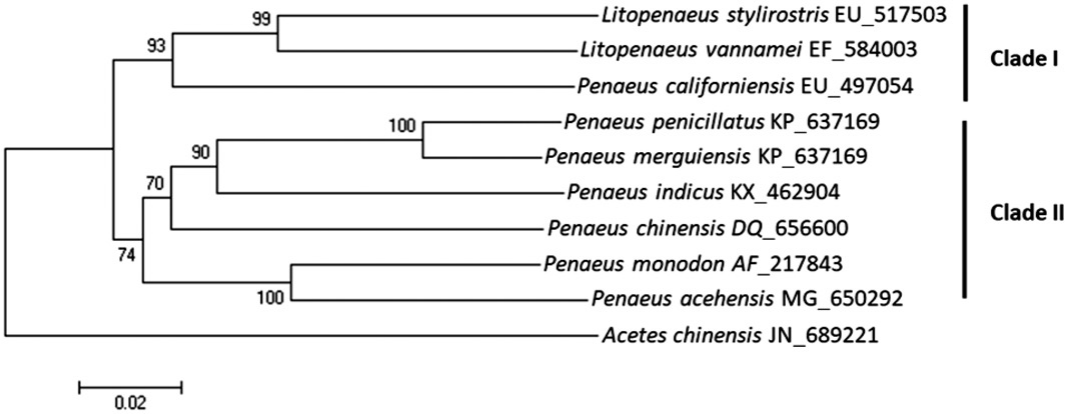
Phylogenetic trees of *Penaeus acehensis*. The phylogenetic tree was constructed using molecular evolutionary genetic analyses (MEGA 6, version 6.0) with the minimum evolutionary algorithm. The evolutionary distance was calculated using Kimura 2 parameter method. Bootstrap replications were 1000. GenBank Accession number for each species was shown in bracket.
